# Age-related changes of the innate immune system of the palatine tonsil in a healthy cohort

**DOI:** 10.3389/fimmu.2023.1183212

**Published:** 2023-06-29

**Authors:** Nico Andreas, Katharina Geißler, Juliane Priese, Orlando Guntinas-Lichius, Thomas Kamradt

**Affiliations:** ^1^ Institute of Immunology, Jena University Hospital, Jena, Germany; ^2^ Department of Otorhinolaryngology, Jena University Hospital, Jena, Germany

**Keywords:** healthy palatine tonsil, blood, myeloid, human, immune system, aging, flow cytometry

## Abstract

Although tonsillectomy is performed frequently, the role of palatine tonsils in life long immune protection or tolerance is still debated and the consequences of their removal for the immune system are of general interest. We analysed the tonsillar myeloid compartment in healthy subjects across a wide range of age (64% male; age range: 3 - 85 years) and compared its composition to the peripheral blood. We could observe a strong accumulation of all granulocyte subsets in the aging tonsil, which was most pronounced for basophils and mast cells. On functional level, an increase of CD163 and CD206 expression among monocytes and an increase of neutrophils expressing the inhibitory FcγRIIb correlated with increasing age. While the age-related shift of the leukocyte composition towards monocytes in blood is not reflected in tonsils, the increasing immunoregulatory phenotype of tonsilar monocytes is potentially counteracting the phenomenon of inflammaging at higher age.

## Introduction

Tonsillectomy, i.e. the removal of the palatine tonsils, still is one of the most frequent surgical procedures (1). The highest prevalence of tonsillectomies is seen in the age group of less than 18 years (2) and decreases with higher age (3). Most of these surgeries are indicated to treat tonsil hyperplasia, recurrent acute tonsillitis, or a peritonsillar abscess (2).

As a part of the peripheral lymphoid system, palatine tonsils are located at the entrance of the upper aerodigestive tract. They are involved in the protection against ingested and inhaled pathogens. Within the tonsils, a network of interactions between lymphocytes and innate immune cells controls the response to various infections. The most represented population are B cells (3, 4), which mainly induce IgG-mediated responses, e.g., against Eppstein Barr virus infections (5). Tonsil-derived B cells exist in distinct activation and maturation states and even contain a newly identified activated CCL3/CCL4 producing B cell subset (6). Tonsillectomy early in life may lead to a lack of differentiation of CD10+ B cells associated with a decrease of antibody switching (7). Besides B cells, T cells are the second most represented immune cell subset and induce various humoral and cell-mediated immune responses, and even oral tolerance (8–10).

It is yet not clear, whether tonsils significantly enhance protection towards infections, or whether a lack of tonsils can be fully compensated. This knowledge is of general importance due to the aforementioned high number of tonsillectomies in infants and juvenile patients leaving them to a potentially life-long impaired immune control.

A lack of tonsils has been associated with higher risk for lower respiratory tract infections (11), higher risk of Hodgkin’s lymphoma (12), or an enhanced susceptibility for autoimmune diseases (13). In contrast to these reports, a meta-analysis of many studies did not reveal any significant immune defect induced by tonsillectomy (14). In line with that, humoral immune responses and immunological memory were not affected upon tonsil resection (15, 16). These contradictive observations are even more enigmatic, since the tonsils are composed of all cellular compartments necessary to induce a full immune response, e.g. antigen-presenting cells, T cells, B cells and various accessory cells (8). Even more intriguingly, in a 20-year follow up study, the prevalence of chronic diseases was increased after tonsillectomy (17). This is supported by a recent study of more than a million of children in Denmark, who underwent tonsillectomy within the first 9 years of life and developed significantly higher risks for respiratory, infectious or asthmatic diseases (18).

Depending on the infection scenario, we could identify various, functionally distinct T cell subpopulations in patients with recurrent acute tonsillitis (19). Apart from that, little is known about age-associated changes in the tonsillar innate immune system from early infants to elder people. In the presented work, we analysed non-inflamed palatine tonsils derived from surgeries for tonsillar hyperplasia or biopsies of healthy tonsils. This allowed us to investigate the aging of the immune system in a peripheral secondary immune compartment under healthy conditions. While some age-related alterations in the blood could be detected in the tonsils, others did not translate.

## Materials and methods

### Patients and tonsillectomy

To analyze the age-related changes in the cellularity of innate immune cells in palatine tonsils, we selectively collected healthy tonsillar tissue and blood from patients at the age of 3 to 85 years (**Table 1**). The ethical review committee from the medical faculty of the Friedrich-Schiller-University Jena approved the study protocol (No. 3972-01/14), which followed the ethical guidelines of the 1975 Declaration of Helsinki. From all patients a written consent was obtained before enrolment in the study. Tissue specimens were obtained mainly by tonsillotomy in the very young study participants undergoing tonsil surgery due to sleep disorders. During this procedure a tissue biopsy of 5 - 15 mm^3^ was obtained. Small 5 mm^3^ punch biopsies were taken in elder participants. The elder patients were recruited from cohorts admitted for head and neck surgery excluding all individuals with a tonsillar disease or its treatment of any other tonsils (adenoid, tubal, lingual). Up to 27 mL peripheral blood was acquired from all probands and was matched to the specimens. Exclusion criteria were acute tonsillitis within the last 12 months, steroidal or other immunosuppressive therapy, cancer or history of cancer, severe chronic diseases in medical history, or a therapy with anticoagulants or coagulation values lying under standard average values. All surgeries were performed in the department of otorhinolaryngology of the Jena University Hospital, Germany. The patients were recruited between July 2019 and June 2021. 23 male and 13 female patients of various ages were included (**Table 1**).

**Table 1:** Patients’ characteristics for the different age cohorts.

	Total	Age 0-18 years	Age 18-40 years	Age 41-70 years	Age >70 years
	N	N	N	N	N
Gender					
male female	23 13	7 3	5 4	5 5	6 1
	Mean ± SD	Mean ± SD	Mean ± SD	Mean ± SD	Mean ± SD
Age, years	40 ± 28.2	5.7 ± 3.7	29 ± 6	56 ± 8.7	80.1 ± 3.2
Age range, years	3-85	3-14	18-36	42-70	75-85

SD, standard deviation.

### Preparation of cells suspension

Whole blood samples were incubated with ery lysis buffer (H_2_O, 0.15mM NH_4_Cl, 1mM KHCO_3_, 0.1mM Na_2_EDTA, pH 7.4) for at RT 5 min. Reaction was stopped by addition of PBA-E (PBS, 5 mg/mL, BSA, 10 mM NaN3, 2 mM EDTA) and centrifuged at 300 x g for 10 min. If pellet remained red, the procedure was repeated. Tonsil tissue was minced and digested in 1 mg/mL Collagenase IV (Worthington, USA) gently stirred at 37°C for 60 min, and subsequently pressed through a 70 µM cell strainer (Thermo Fisher Scientific) to obtain single cell suspension.

### Flow cytometric analysis

2 x 10^6^ cells were resuspended in 1 mg/mL beriglobin in PBA-E, incubated for 5 min and subsequently stained according to manufacturers recommendation with following antibodies in PBA-E in darkness at 4°C for 20 min. Set 1: CD45 V500 (clone HI30, BD Biosciences), CD15 BV605 (clone W6D3, BD Biosciences), CD14 FITC (clone M5E2, BD Biosciences), CD163 PE (clone GHI/61, BD Biosciences), CD32b PE/Cy7 (clone FUN-2, Biolegend), CD16 PerCP-Cy5.5 (clone 3G8, BD Biosciences), CD206 Alexa Fluor 700 (clone 15-2, Biolegend) and CD64 APC-H7 (clone 10.1, BD Biosciences); Set 2: CD45 V500 (clone HI30, BD Biosciences), FceRI BV605 (clone AER-37 (CRA1), BD Biosciences), CD141 FITC (clone JAA17, ThermoFisher (eBioscience)), CD303 PE/Cy7 (clone 201A, Biolegend), CD1c PerCP-Cy5.5 (clone F10/21A3, BD Biosciences), CD11c APC (clone N418, Biolegend), HLA-DR Alexa Fluor 700 (clone G46-6 (L243), BD Biosciences), CD117 APC/Cy7 (clone 104D2, Biolegend). Cells were washed with PBA-E and analysed on a FACS Canto-Plus flow cytometer (Becton Dickinson). Prior analysis, 1 µg/mL DAPI (4′,6-diamidino-2-phenylindole, Cell Signaling Technology) was added to each sample. Data were analysed with FlowJo V10.7 (BD).

### Statistical test

Trend lines were calculated with EXCEL (Microsoft Office 365, Version 2209). Zero values, which could not have been displayed in logarithmic diagrams, were included for statistical analysis. Statistical test was done with Spearman Rank Order Correlation using Sigmaplot V14.5 (Systat Software, Germany). *p<0.05, **p<0.01, ***p<0.001, ***^n.s.^*** non-significant

## Results

### Accumulation of neutrophilic and eosinophilic granulocytes in aging tonsils

**Figure 1:**
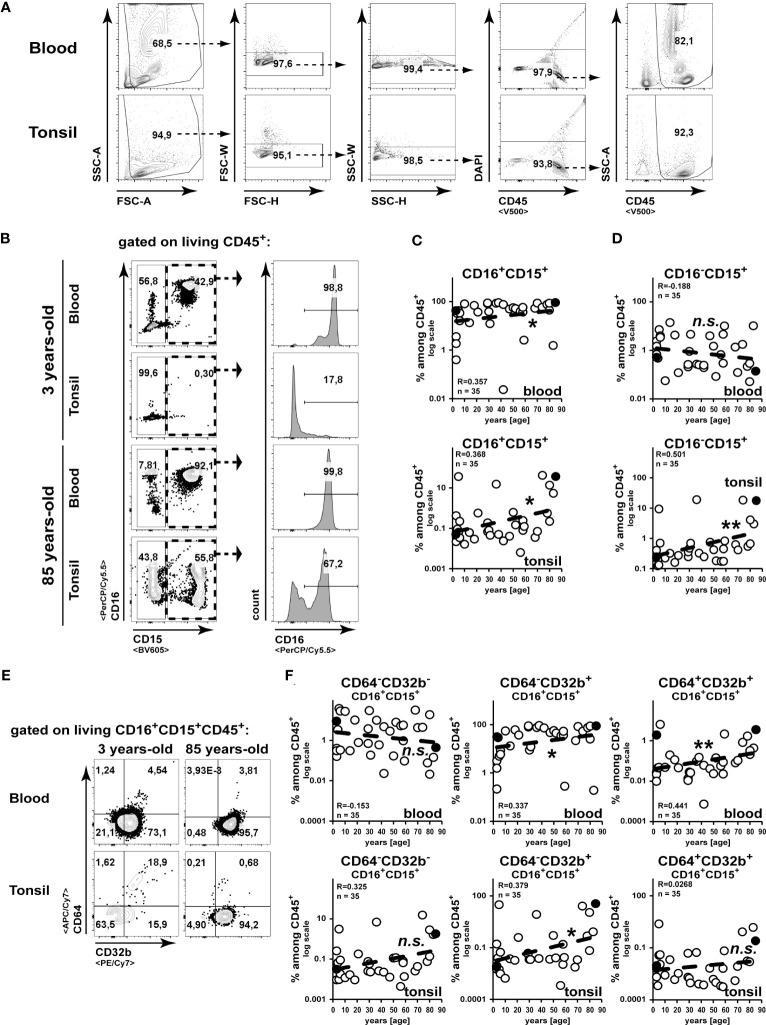
Accumulation of granulocytes in the tonsils during aging. Cell suspensions from blood and palatine tonsils of patients at different ages were analysed by flow cytometry. **(A)** Gating strategy for CD45^+^ cells is shown. **(B–D)** CD16^+^CD15^+^ or CD16^-^CD15^+^ among CD45^+^ cells were analysed. Representative gating strategy is shown in **(B)** and x/y diagrams summarize the data of CD16^+^CD15^+^ **(C)** or CD16^-^CD15^+^ **(D)** cells among CD45^+^ cells from blood (upper panels) or tonsils (lower panels) **(D)**. **(E, F)** CD64 or CD32b expressing CD16^+^CD15^+^CD45^+^ cells were analysed by flow cytometry. Representative FACS plots are shown in **(E)** and summarized data for the indicated populations are shown for blood (upper panels) or tonsils (lower panels) in **(F)**. Data points representing the dot plot examples are marked as black circles in the diagrams. Trend line is represented by the dashed line in the diagrams. ^n.s.^not significant, *p<0.05, **p<0.01.

While the age-related alteration of lymphocyte frequencies and function has already been described in detail (20, 21), this study analysed the age-related composition of the innate immune compartment by flow cytometry. We identified hematopoietic cells by the expression of CD45 as shown in the representative gating strategy (**Figure 1A**). To identify neutrophils and eosinophils, we separated cells expressing the common granulocyte marker CD15 by their CD16 expression into CD16^+^CD15^+^ neutrophils and CD16^-^CD15^+^ eosinophils (22) (**Figure 1B**). In line with former studies (23), we measured an age-associated increase in the frequencies of blood neutrophils (**Figure 1C**), while eosinophils did not change among blood leukocytes (**Figure 1D**). Interestingly, both subsets of granulocytes did accumulate among CD45^+^ cells in aging tonsils (**Figures 1C, D**). Although CD32b^+^ neutrophils increased in the blood CD45^+^ compartment upon aging, of them only CD64^-^CD32b^+^ cells accumulated in aging tonsils (**Figures 1E, F**).

### Mast cells and basophils accumulate in tonsils during aging

**Figure 2:**
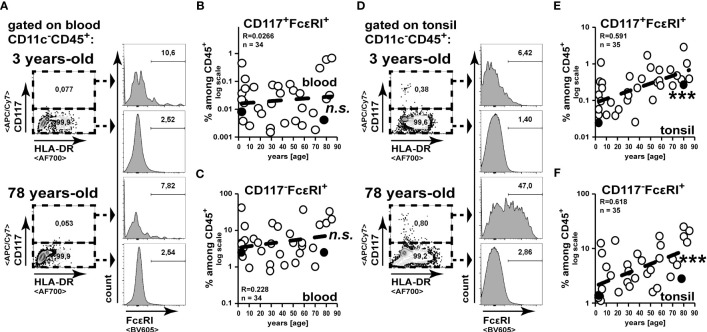
Mast cell and basophil frequencies increase in the tonsils during aging. Cell suspensions from blood **(A–C)** and palatine tonsils **(D–F)** of subjects at different ages were analysed for CD117^-^FcεRI^+^ and CD117^+^ FcεRI^+^ cells among CD45^+^ cells. **(A, D)** Representative gating analysis to identify mast cells and basophils is shown. **(B, C)** CD117^+^FcεRI^+^ **(B)** and CD117^-^FcεRI^+^ **(C)** among CD45^+^ cells in blood are summarized in x/y diagrams. **(E, F)** CD117^+^FcεRI^+^ **(E)** and CD117^-^FcεRI^+^ **(F)** among CD45^+^ cells in tonsils are summarized in x/y diagrams. Data points representing the gating examples are marked as black circles in the diagrams. Trend line is represented by the dashed line in the diagrams. ^n.s.^not significant, ***p<0.001.

To analyze the age-associated alteration of frequencies of basophils or mast cells, we analysed CD117^-^FcεRI^+^ or CD117^+^ FcεRI^+^ cells, respectively (**Figure 2**). While among blood leukocytes CD117^-^FcεRI^+^ basophils have been clearly detectable, CD117^+^ FcεRI^+^ mast cells were scarce (**Figures 2A–C**). Confirming previous studies (23), we did not observe any enrichment of basophils or mast cells among blood leukocytes in elder individuals (**Figures 2B, C**). In contrast to this, we did observe a strong increase of basophils and mast cells in tonsils of elder individuals (**Figures 2E, F**). Collectively, we observed an overall increase of granulocytes in tonsils of healthy individuals with increasing age, which could not be correlated to their frequencies in peripheral blood.

### Accumulation of classical monocyte/macrophage subsets in the aging tonsils

As commonly accepted (24), monocytes can by discriminated into distinct major subsets according to their expression of CD14 and CD16. We analysed the age-related changes of the composition of human tonsil monocytes/macrophages according to this nomenclature (**Figure 2**). Surprisingly, we could detect two populations of intermediate CD14+CD16+ monocytes and analysed them separately (**Figure 2**).

**Figure 3:**
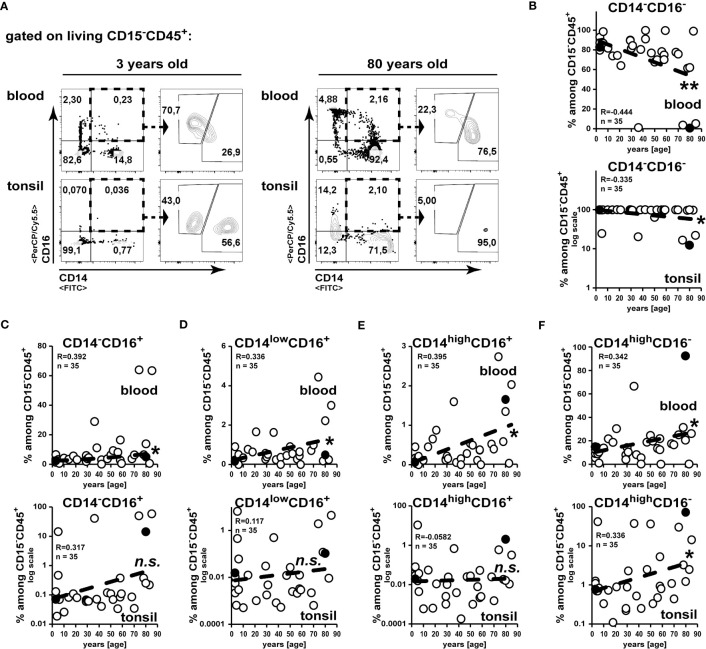
Increased accumulation of classical CD16^+^CD14^high^ macrophages in tonsils of elder subjects. Cell suspensions from blood and palatine tonsils of subjects at different ages were analysed for expression of CD14 and CD16 among CD15^-^CD45^+^ cells. **(A)** Gating strategy to analyze CD14 and CD16 expression among CD15^-^CD45^+^ leukocytes is shown. **(B–F)** Data of subpopulations among CD15^-^CD45^+^ cells are summarized in x/y diagrams for blood (upper panels) or palatine tonsils (lower panels) for: CD14^-^CD16^-^ **(B)**, CD14^-^CD16^+^ **(C)**, CD14^low^CD16^+^ **(D)**, CD14^high^CD16^+^ **(E)** and CD14^high^CD16^-^ **(F)** cells. Data points represented by the dot plot examples are marked as black circles in the diagrams. Trend line is represented by the dashed line in the diagrams. ^.^not significant, *p<0.05, **p<0.01.

In line with the age-related drop of T and B cells in the blood (17), we observed a reduction in frequencies of CD14^-^CD15^-^CD16^-^ leukocytes (**Figure 3A, B**). Whereas among blood CD15^-^CD45^+^ non-granulocytes all monocytes/macrophage subsets increased correlating with age, in tonsils only CD16^+^CD14^high^ classical macrophages were increased in elder subjects (**Figures 3A, C–F**).

### Monocytes/macrophages shift to express CD206 and CD163 in aging tonsils.

**Figure 4:**
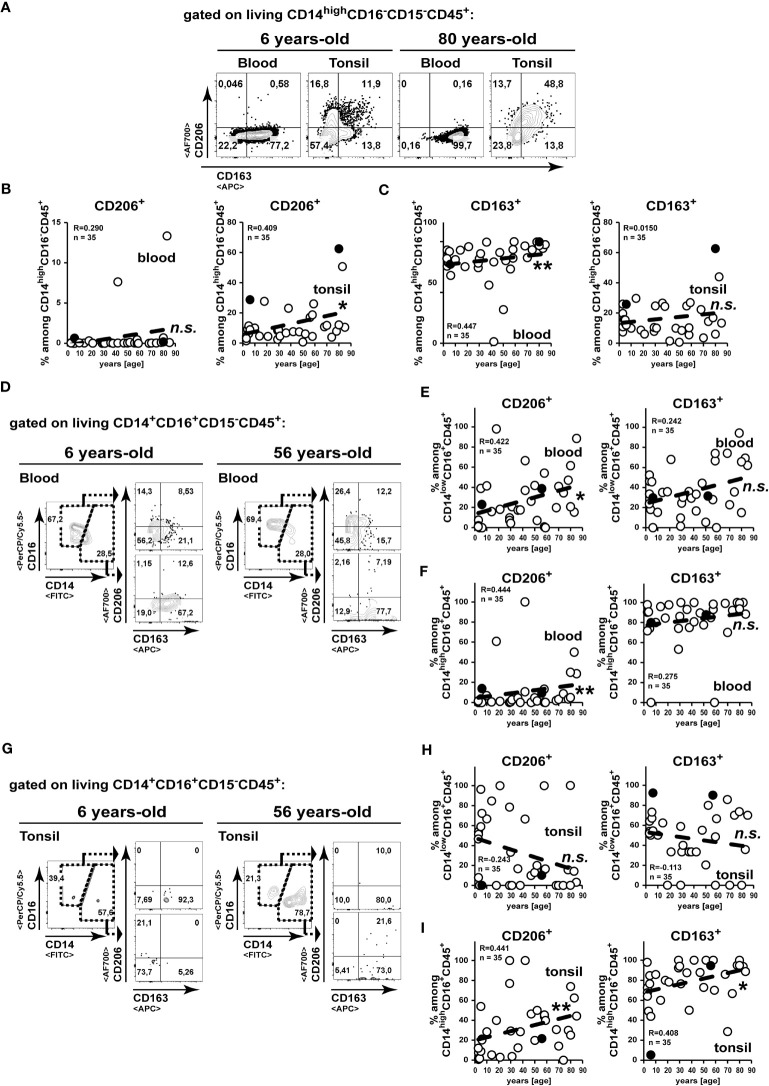
CD206 expressing classical monocytes/macrophages accumulate in aged tonsils. **(A–C)** CD14^high^CD16^-^CD15^-^CD45^-^ classical monocyte/macrophages from blood and palatine tonsils were analysed for expression of CD206 and CD163. Representative FACS plots for CD206 and CD163 expressions are shown **(A)**. CD206 **(B)** and CD163 **(C)** expression levels are summarized among CD14^high^CD16^-^CD15^-^CD45^+^ cells in blood (left) and tonsils (right). **(D-I)** CD14^+^CD16^+^CD15^-^CD45^+^ monocyte/macrophages from blood **(D–F)** and tonsils **(G–I)** were separated into CD14^low^CD16^+^CD15^-^CD45^-^ **(E, H)** and CD14^high^CD16^+^CD15^-^CD45^-^ **(F, I)** cells as shown by representative gating strategies **(D, G)**. Expression frequencies of the indicated marker among the subpopulations are summarized in x/y diagrams. Data points represented by the FACS plot examples are marked as black circles in the diagrams. Trend line is represented by the dashed line in the diagrams. ^n.s.^not significant, *p<0.05, **p<0.01.

To gather a more detailed insight on functional subsets of monocytes/macrophages, we analysed the expression of the C-type lectin CD206 and the scavenger receptor CD163 (**Figures 4A, D, G**). On peripheral blood CD14^+^CD16^-^CD15^-^CD45^+^ classical monocytes, the expression of CD206 appeared scarcely and was not changed in elder subject (**Figure 4B**), whereas we detected high amounts of a CD163^+^ subset, which did significantly increase upon aging (**Figure 4C**). In contrast to this, we measured accumulated CD206^+^ classical monocytes in aged tonsils, whereas CD163 expression remained unchanged in tonsils of elder subjects (**Figures 4B, C**). Among the intermediate blood CD14^+^CD16^+^ monocytes/macrophages we could detect increased frequencies of CD206 expression in aged individuals, while CD163 remained unaltered (**Figures 4E, F**). However, within aging tonsils CD206 and CD163 were not increased on CD14^low^CD16^+^ intermediate monocytes/macrophages (**Figure 4H**), whereas these markers were expressed at increasing levels correlating with age on tonsillar CD14^high^CD16^+^ intermediate monocytes (**Figure 4I**).

Collectively, during age classical and intermediate monocytes/macrophages with enhanced expression of CD206 accumulate in tonsils. While for the classical monocytes/macrophages, the enhanced tonsillar frequencies were not observed in the blood stream, the increase of CD206^+^ intermediate monocyte/macrophage frequencies in the tonsils could result of an equilibrium from increased blood levels.

### CD1c expressing type-1 classical DCs increase in the aging tonsil

**Figure 5:**
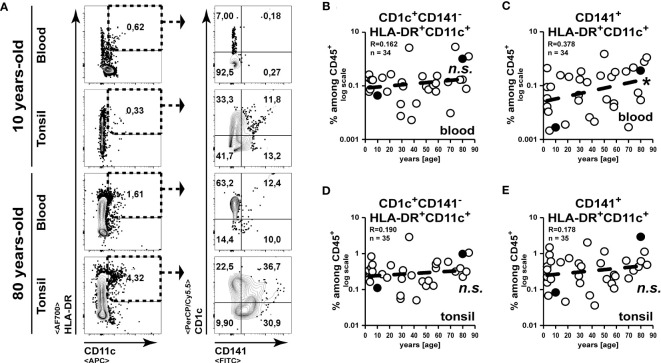
DC frequencies remain unaltered in aged tonsils. Classical DCs in blood and tonsils were identified by the expression of HLA-DR and CD11c in combination with CD141 or CD1c. **(A)** Representative FACS plots for the gating strategy are shown. **(B–E)** Frequencies of CD1c^+^CD141^-^CD11c^+^ **(B, D)** or CD141^+^ CD11c^+^ **(C, E)** cells are summarized in the x/y diagrams for blood **(B, C)** and tonsils **(D, E)**. Data points represented by the FACS plot examples are marked as black circles in the diagrams. Trend line is represented by the dashed line in the diagrams. ^n.s.^not significant, *p<0.05.

Comparable to murine CD8α^+^ and CD11b^+^ DC subsets, human classical DCs can be separated into CD141^+^ (DC1) and CD1c^+^ (DC2) subsets, respectively (24, 25). While CD11c is not exclusively expressed on human dendritic cells but is shared with human monocytes (26), we analysed cells expressing a combination of CD11c with CD141 (27) or with CD1c (28) to sufficiently identify the major cDC subsets (**Figure 5A**). While CD1c^+^CD141^-^ DC2 subset frequencies remained unaltered, we could detect an increase of CD141^+^ DC1 frequencies among blood cells upon aging (**Figures 5B, C**). However, the CD1c^+^CD141^-^ DC2 subset frequencies remained unaltered in the tonsils (**Figure 5D**) and an age-related increase of CD141^+^ DC1 frequencies did not occur in tonsils of elder subjects as observed in blood (**Figure 5E**). Within a few samples, we could analyse plasmacytoid dendritic cells (pDCs) by the expression of CD303 (29) and we detected a strong age-related decrease of pDCs among blood leukocytes (**Supplement-Figures 1A, B**). However, a comparable drop was not detected in the tonsils of elder subjects (**Supplement-Figures 1A, B**). Collectively, while DC frequencies were partially altered among blood cells upon aging, these changes did not affect DC frequencies within tonsil.

## Discussion

In the presented work, we analysed the myeloid cell composition among CD45^+^ PBMCs and among CD45^+^ hematopoietic tonsillar cells of up to 36 subjects of various ages. Correlation of measured frequencies to age of the donors revealed an increased skewing towards myeloid differentiation at the cost of lymphopoiesis, which is in line with previous findings (30). Upon aging, the individuals have increasing neutrophils with a reduced phagocytic ability and a decreased bactericidal activity (31).

Previous reports have indicated that in infants blood neutrophil expression of CD64 decreases in infants with increasing age (32). In line with this, pre-term infants show an even higher frequency of CD64^+^ expressing neutrophils (33). However, the aforementioned reports describe subjects until the first year of life, whereas we detected increasing frequency of CD64^+^ expressing neutrophils beyond that age, albeit at very low frequencies. This did not translate on to neutrophils in tonsils, where we detected an enrichment of CD64^-^ negative neutrophils. However, we could detect the increased frequencies of CD64^-^CD32b^+^ neutrophils among blood as well as on tonsillar leukocytes. Stervbo et al. observed a significant increase in the frequency of transitional and CD14^low^CD16^++^ non-classical monocytes in the elderly compared with the young (34). This observation is consistent with previous studies showing an age-dependent decrease of classical monocytes, while minor subsets increased correspondingly (35, 36).

The major subsets of T and B cells strongly decrease with age in the blood, whereas other granulocytes and monocytes remain unchanged (37). A less pronounced drop in neutrophil numbers leads thereby to a virtual increase in neutrophil frequencies among leukocytes (37). T cells are increasing with age up to the age of 40, and thereafter decrease again (20). While aging, tonsillar T cell compositions shift from CD8^+^ towards CD4^+^ enrichment, and B lymphocytes drop (20). Age-related changes are preferentially investigated in blood due to its availability and accessibility. T cells frequencies in blood increase with age, while B cells strongly decrease (23). NK cell numbers in blood increase with age, but also lose cytotoxic and cytokine-producing potential (21, 38).

These age-related events together with the declining functionality of neutrophils might impair the role of tonsilar tissue as a gate-keeping immune center for microorganism/biochemical substances accessing the body *via* the oral cavity to induce immune responses against respiratory infections.

The presented study has relevant limitations. The tonsillar samples consisted of 10 individuals per age cohort. This clearly limits the generalizability of the findings. To get representative data of a normal population adequately representing the distribution of characteristics of each age group, a further study with large sample size is needed. Especially, the group of minors must be further divided in more subgroups to better distinguish effect of immune system maturation from age changes in the adult tonsillar system.

Overall, there is not much data on healthy tonsil tissue. Apparently, the immune system in normal tonsil tissue is very different from that in recurrent acute tonsillitis, because here the situation is partially different, e.g., sclerotic tonsils have fewer neutrophils, more often bacteremia after tonsillectomy and this can be caused by a restricted immune system (39).

Collectively, our study demonstrates that tonsils do not completely reflect the age-related leukocyte changes in the blood. In contrast to the blood, tonsillar monocytes acquire an alternatively activated phenotype and potentially balance increasing inflammatory processes upon aging. In line with this, the increased frequency of mast cells might support this tipped immune balance and thereby, counteract a detrimental overshooting cellular immune responses associated with autoimmunity. Thus, our results argue for a more careful indication of tonsillectomy and should sensitize for a potential bias of tonsillectomized individuals towards autoimmune syndromes.

## Data availability statement

The raw data supporting the conclusions of this article will be made available by the authors, without undue reservation.

## Ethics statement

The studies involving human participants were reviewed and approved by The ethical review committee from the medical faculty of the Friedrich-Schiller-University Jena approved the study protocol (No. 3972-01/14), which followed the ethical guidelines of the 1975 Declaration of Helsinki. Written informed consent to participate in this study was provided by the participants’ legal guardian/next of kin.

## Author contributions

NA, KG, OG-L and TK had full access to all the data in the study and take responsibility for the integrity of the data and the accuracy of the data analysis. Study concept and design: TK, OG-L. Acquisition of data: NA, KG, JP. Analysis and interpretation of data: NA, KG, OG-L, TK. Drafting of the manuscript: NA, KG. Critical manuscript revision and additional important intellectual content, data interpretation: NA, KG, OG-L, TK. Statistical Analyses: NA. All authors contributed to the article and approved the submitted version.

## Supplementary material

Supplementary Figure 1 Strong age-dependent drop of blood pDC frequencies is not reflected in tonsil. pDCs in blood and palatine tonsils were identified by the expression of CD303 in absence of CD11c. **(A)** Representative FACS plots for the gating strategy are shown. **(B, C)** Frequencies of CD303^+^CD11c^-^ cells among CD45^+^ are summarized in the x/y plots for blood **(B)** and tonsils **(C)**. Data points represented by the dot plot examples are marked as filled circles in the diagrams. Trend line is represented by the dashed line in the diagrams. ^n.s.^not significant, ***p<0.001.
