# Supplementary material for: Age-related changes of the innate immune system of the palatine tonsil in a healthy cohort
Source: Front Immunol. 2023 Jun 29;14:1183212. doi: 10.3389/fimmu.2023.1183212 (PMC10344772; doi:10.3389/fimmu.2023.1183212)
Supplement: Supplementary Figure 1 — Strong age-dependent drop of blood pDC frequencies is not reflected in tonsil. pDCs in blood and palatine tonsils were identified by the expression of CD303 in absence of CD11c. (A) Representative FACS plots for the gating strategy are shown. (B, C) Frequencies of CD303+CD11c- cells among CD45+ are summarized in the x/y plots for blood (B) and tonsils (C). Data points represented by the dot plot examples are marked as filled circles in the diagrams. Trend line is represented by the dashed line in the diagrams. n.s.not significant, ***p<0.001. [file DataSheet_1.pdf]

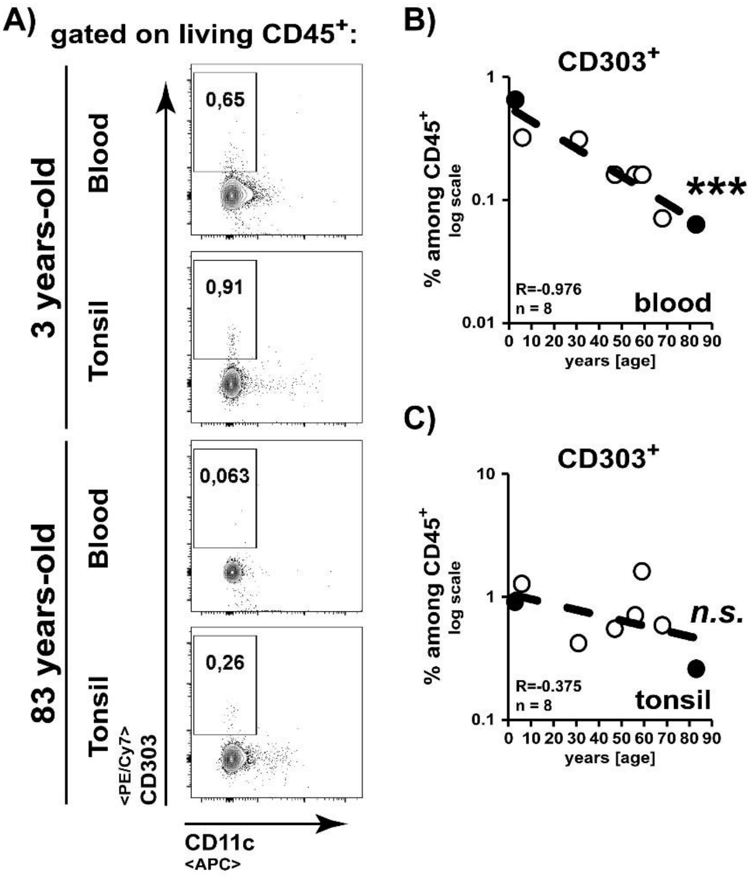

Supplement-Figure 1: Strong age-dependent drop of blood pDC frequencies is not reflected in tonsil. pDCs in blood and palatine tonsils were identified by the expression of CD303 in absence of CD11c. **A)** Representative FACS plots for the gating strategy are shown. **B, C)** Frequencies of $CD303^{+}CD11c^{-}$ cells among $CD45^{+}$ are summarized in the x/y plots for blood (B) and tonsils (C). Data points represented by the dot plot examples are marked as filled circles in the diagrams. Trend line is represented by the dashed line in the diagrams. $^{n.s.}$*not significant*, ****p<0.001*
